# EN1 promotes lung metastasis of salivary adenoid cystic carcinoma by regulating the PI3K-AKT pathway and epithelial-mesenchymal transition

**DOI:** 10.1186/s12935-024-03230-7

**Published:** 2024-01-30

**Authors:** Yajuan Cui, Ye Zhang, Yuping Liu, Zheng Zhou, Lijing Zhu, Chuan-Xiang Zhou

**Affiliations:** Department of Oral Pathology, Peking University School and Hospital of Stomatology & National Center of Stomatology & National Clinical Research Center for Oral Disease & National Engineering Research Center of Oral Biomaterials and Digital Medicine Devices, 22 South Avenue Zhongguancun, Haidian District, Beijing, 100081 PR China

**Keywords:** Salivary adenoid cystic carcinoma, Engrailed 1, Metastasis, Methylation, Epithelial-mesenchymal transition

## Abstract

**Background:**

Engrailed homeobox 1 (*EN1*) is a candidate oncogene that is epigenetically modified in salivary adenoid cystic carcinoma (SACC). We investigated the expression of *EN1* in SACC tissues and cells, *EN1* promoter methylation, and the role of EN1 in tumour progression in SACC.

**Methods:**

Thirty-five SACC samples were screened for key transcription factors that affect tumour progression. In vitro and in vivo assays were performed to determine the viability, tumorigenicity, and metastatic ability of SACC cells with modulated EN1 expression. Quantitative methylation-specific polymerase chain reaction analysis was performed on SACC samples.

**Results:**

EN1 was identified as a transcription factor that was highly overexpressed in SACC tissues, regardless of clinical stage and histology subtype, and its level of expression correlated with distant metastasis. EN1 promoted cell invasion and migration through epithelial-mesenchymal transition in vitro and enhanced SACC metastasis to the lung in vivo. RNA-seq combined with in vitro assays indicated that EN1 might play an oncogenic role in SACC through the PI3K-AKT pathway. *EN1* mRNA levels were negatively correlated with promoter hypermethylation, and inhibition of DNA methylation by 5-aza-dC increased EN1 expression.

**Conclusions:**

The transcription factor EN1 is overexpressed in SACC under methylation regulation and plays a pivotal role in SACC progression through the PI3K-AKT pathway. These results suggest that *EN1* may be a diagnostic biomarker and a potential therapeutic target for SACC.

**Supplementary Information:**

The online version contains supplementary material available at 10.1186/s12935-024-03230-7.

## Introduction

Salivary adenoid cystic carcinoma (SACC) is a rare malignant tumour that accounts for 1% of malignant neoplasms of the head and neck and 10% of salivary gland malignancies. The age at onset is usually between 50 and 60 years, with no difference between males and females [1]. SACC is characterised by indolent growth, perineural invasion, and hematogenous metastases, most commonly in the lungs [2].

The role of transcription factors in tumours is increasingly recognized. Transcription and regulatory factors account for 19% of the known oncogenic genes in the cancer gene network [3]. The transcription factor MYB may serve as a diagnostic marker for SACC [4]. In this study, we identified engrailed homeobox 1 (EN1) as a key transcription factor in SACC. EN1 is a member of the homeobox family and plays a significant role in tumorigenesis. In vertebrates, two engrailed homeobox genes have been discovered, EN1 and EN2 [5, 6]. In humans, aberrant EN1 expression is associated with the pathogenesis of many types of tumours, including breast cancer [7], prostate cancer [8], colorectal cancer [9], glioma [10], and nasopharyngeal carcinoma [11]. However, the role of EN1 in SACC remains unclear.

Here, we aimed to investigate the role of EN1 in tumour proliferation, invasion, and metastasis of SACC, analyse the correlation between EN1 expression and the clinicopathological characteristics of the patients with SACC, and explore the possible mechanism underlying EN1 overexpression and its role in SACC.

## Patients and methods

### Patients and tissue samples

SACC and other salivary gland neoplasms from 2001 to 2019 were obtained from the files of the Peking University School and Hospital of Stomatology. Slides were reviewed by two senior pathologists to confirm histological diagnosis and grading. Paraffin sections from 100 patients with SACC and 16 patients with other salivary gland neoplasms (three salivary ductal carcinomas, five acinic cell carcinomas, five pleomorphic adenomas and three adenocarcinomas, not otherwise specified (NOS)) were immunohistochemically stained. Frozen tissues from 35 SACC patients were used for paired-tissue RNA sequencing (RNA-seq), and frozen tissues from 22 SACC patients were used for methylation detection. Patient follow-up data, as of December 2022, were obtained by reviewing medical records, telephone interviews, and/or clinical examinations. This study was approved by the Institutional Ethics Committee of the university (2021-NSFC-43).

### Immunohistochemistry

Formalin-fixed paraffin-embedded tumour tissues were cut into 4-µm-thick serial sections, processed, and analysed as previously described [12]. Primary antibodies to EN1 (1:100; Atlas Antibodies, Stockholm, Sweden) were used for immunostaining. Two independent, blinded observers semi-quantitatively analysed the ratio of positive cells to determine the immunostaining score. The percentage of positive cells was scored as follows: 0, up to 2%; 1, 2–25%; 2, 25–50%; 3, 50–75%; and 4, > 75%. Only nuclear staining was evaluated as positive. Samples with a score of 0, 1, or 2 were defined as “low EN1 expression,” whereas samples with a score of 3 or 4 were defined as “high EN1 expression” (overexpression).

### Cell lines and cell culture

The SACC cell lines SACC-83 and SACC-LM were obtained from the Department of Central Laboratory, Peking University School and Hospital of Stomatology. The authenticity and purity of cells were confirmed by short tandem repeat analysis [13, 14].

### Lentivirus construction and infection

The coding sequence of *EN1* mRNA (NM_001426.4) was synthesized and cloned into the pLenti6.3 lentivirus overexpression vector; the *EN1*-overexpressing and control lentiviruses were constructed by Shanghai Hanbio Technology Company. After packaging, the HBLV-h-*EN1*-3xflag-ZsGreen-PURO lentivirus and the negative control HBLV-ZsGreen-PURO lentivirus were used to infect SACC-83 cells to construct *EN1*-overexpressing (EN1-ove) and negative control SACC-83 (83 control) cells. *EN1* knockout (L26436) and negative control (L00015) CRISPR-Cas9 lentiviruses were purchased from Beyotime (Shanghai, China). After packaging, pLenti-EN1-sgRNA-PURO lentivirus and negative control pLenti-control-sgRNA-PURO lentivirus were used to infect SACC-LM cells for the construction of *EN1* knockdown and negative control SACC-LM (LM control) cells.

### RNA preparation and qPCR

RNA was extracted using TRIzol (Invitrogen Life Technologies, Carlsbad, CA, USA) following the manufacturer’s instructions. The extracted RNA was reverse-transcribed into cDNA using a cDNA Reverse Transcription Kit (Takara, Beijing, China). qPCR was conducted with FastStart Universal SYBR Green Master (ROX) Reagent (Roche, Basel, Switzerland) on an ABI Prism 7500 Real-Time PCR System (Applied Biosystems, CA, USA). β-actin mRNA was used for normalization. The 2^−ΔΔCT^ method was used to quantify the relative gene expression levels. Primer sequences (forward and reverse) were as follows: *EN1*: 5′-GCACACGTTATTCGGATCG-3′, 5′-GCTTGTCCTCCTTCTCGTTCT-3′; and *β-actin*: 5′-CTCCATCCTGGCCTCGCTGT-3′, 5′-GCTGTCACCTTCACCGTTCC-3′.

### DNA extraction and bisulfite treatment

Genomic DNA was extracted from frozen tumour and control samples using The Fast DNA Tissue Kit (Qiagen, Hilden, Germany) following the manufacturer’s protocol. The EZ DNA Methylation-Gold Kit (ZYMO Research, CA, USA) was used to convert unmethylated cytosines in the genomic DNA to uracil following the manufacturer’s instructions.

### Quantitative methylation-specific PCR and Sanger sequencing

Quantitative methylation-specific PCR (qMSP) was performed as previously described [15–17]. The following thermocycling conditions were used: initial denaturation at 95 °C for 10 min; 45 cycles of 95 °C for 20 s, 58 °C for 30 s, and 72 °C for 30 s; melting curve analysis at 95 °C for 15 s, 58 °C for 1 min, and 60 °C for 1 min; and a final cooling stage at 40 °C for 10 min. The primer sequences for the *EN1* promoter were forward, 5′-GGGTAGTTTTAGGGTGTTGT-3′ and reverse, 5′-ACTTTCAAAAACCCAATTTATTTTTCACA-3′; and the primer sequences for *β-actin* were forward, 5′-TGGTGATGGAGGAGGTTTAGTAAGT-3′ and reverse, 5′-AACCAATAAAACCTACTCCTCCCTTAA-3′. The percentage of methylated reference (PMR) of *EN1* for each sample was calculated using the 2^−ΔΔCq^ quantification approach, where ΔΔCq = sample DNA (Cq_EN1_-Cq_act_ control)-fully methylated DNA (Cq_EN1_-Cq_act_ control). Sanger sequencing was performed as previously described [18], and C-U conversion was validated by matched normal tissue sequencing.

### Drug treatment

5-Aza-2′-deoxycytidine (5-aza-dC, San Antonio, TX, USA) is a nucleoside analogue that causes hypomethylation of genes by depleting DNA methyltransferase 1 [19]. This demethylation at CpG islands can often reactivate the expression of methylation-silenced genes [20]. SACC-83 cells were treated with 5-aza-dC as described previously [21, 22].

LY294002 was purchased from Selleck Chemicals (Houston, TX, USA). SACC cells overexpressing EN1 and control cells were treated with 20 µM LY294002 for 24 h before biological functional assays. For western blotting, cells were harvested after 48 h.

### Statistical analysis

Data were analysed using SPSS (version 25.0; IBM Corp., Armonk, NJ, USA). For the immunostaining data, Pearson’s chi-square and Pearson’s correlation tests were performed to compare variables between groups. Owing to the skewed distribution of methylation levels, the data were presented as medians (interquartile ranges). Friedman and Wilcoxon nonparametric tests were used to assess differences in methylation between samples. The Mann–Whitney U nonparametric test was used to assess the difference in methylation between groups. Spearman’s rank correlation coefficient was used to assess the correlation between *EN1* methylation and gene expression levels. A two-tailed *P* < 0.05 was considered statistically significant. Figures were plotted using GraphPad Prism 8.0 software (GraphPad Software, Inc., La Jolla, CA, USA). Sequencing results were analysed using the SnapGene software (www.snapgene.com).

## Results

### Identification of differentially expressed transcription factors in SACC

**Fig. 1:**
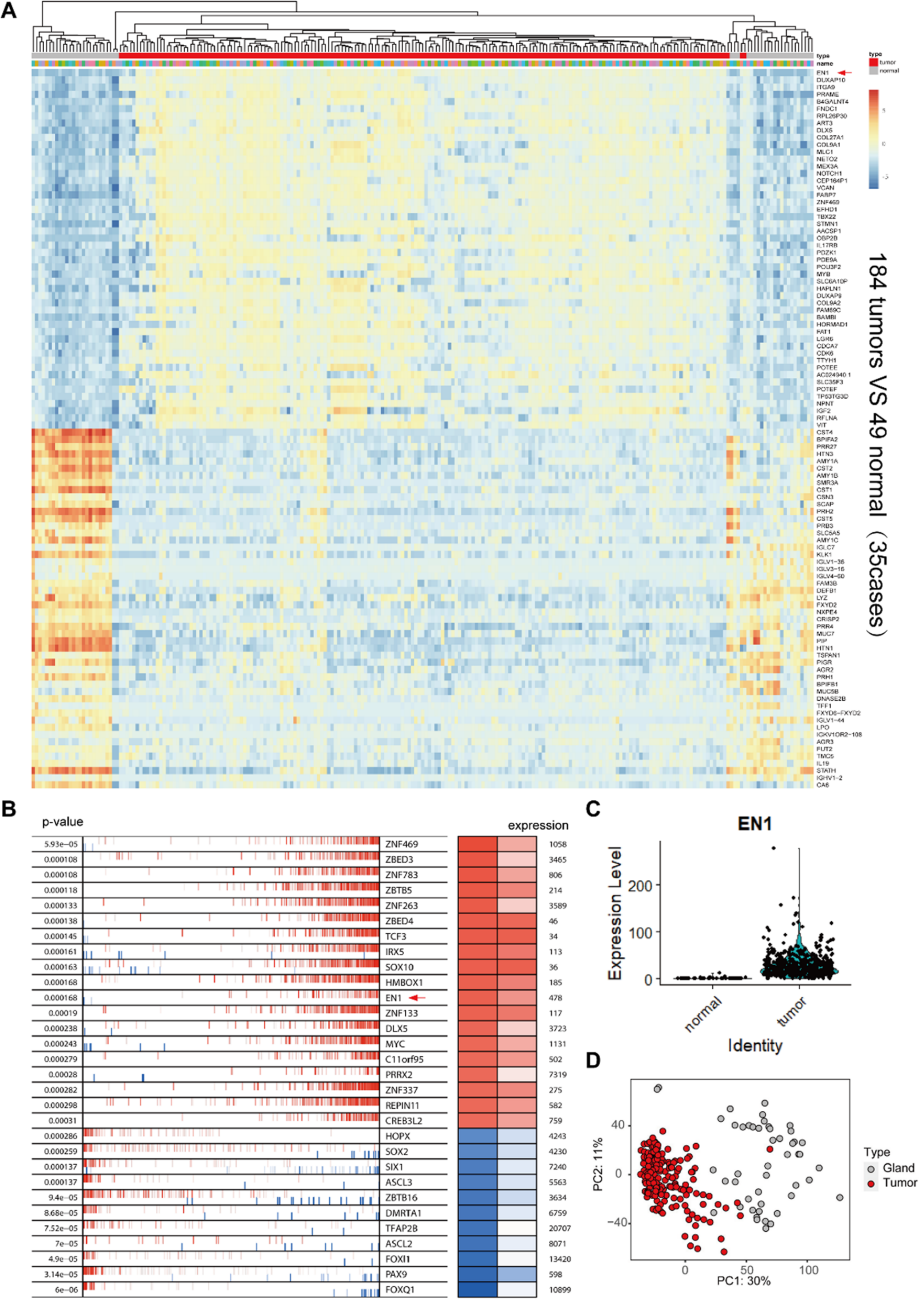
Identification of SACC-specific transcription factors. **A** Heatmap of differentially expressed genes between SACC tumour and normal salivary gland tissues. Tumour samples are shown in red, and normal gland samples are shown in grey. **B** Heatmap of differentially expressed transcription factors between SACC tumour and normal salivary gland tissues. **C** Violin chart depicting *EN1* mRNA expression in SACC tumour and normal salivary gland tissues using RNA-seq data. **D** Principal component analysis of differentially expressed genes between SACC tumour and normal salivary gland tissues. Tumour samples are clustered on the left side (red), and normal samples are clustered on the right side (grey) of the plot

Next-generation RNA-seq was performed on 184 tumour samples and 49 paired normal gland samples from 35 patients with SACC (Additional file 1: Table S1). We identified DEGs between the SACC tumour and normal gland groups **(**Fig. 1A). The results revealed 5,149 DEGs, including 3,979 upregulated DEGs and 1,170 downregulated DEGs (Additional file 2: Table S2). *EN1* showed the most significant difference in expression between tumour and normal tissues (*P*<0.0001).

We identified 19 transcription factors that were significantly overexpressed in SACC tissues compared with normal tissues (Fig. 1B). After comparing the expression levels of the differentially expressed transcription factors and conducting a literature review to determine the biological relevance of the 19 genes, we focused on *EN1*. RNA sequencing data showed that *EN1* mRNA levels in tumour tissues were significantly higher than in control tissues **(**Fig. 1C). The RNA-seq data were subjected to unsupervised principal component analysis (Fig. 1D).

### EN1 is highly expressed in SACC, and its expression correlates with metastasis

**Fig. 2:**
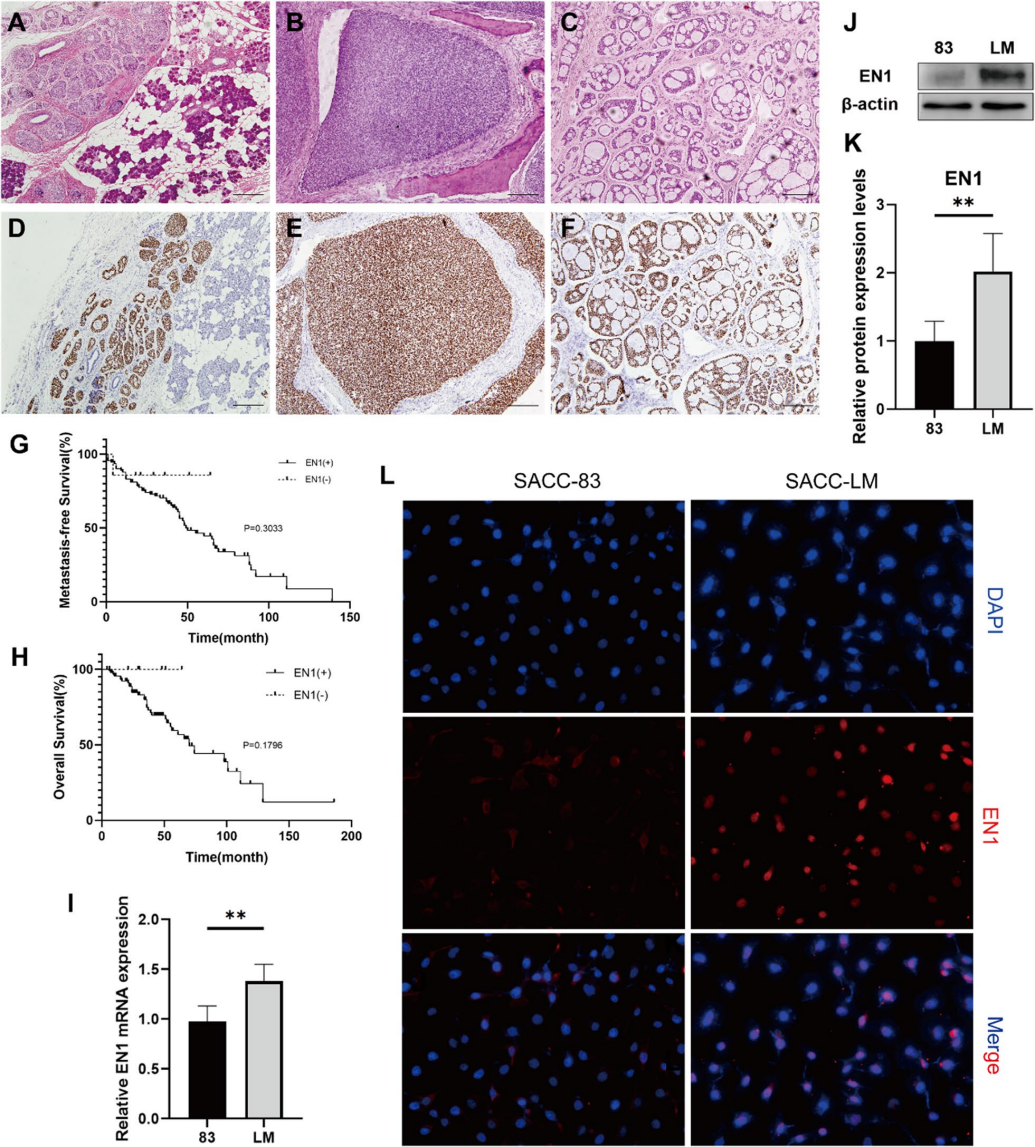
EN1 is highly expressed in SACC, which is correlated with metastasis prognosis. **A–F** Haematoxylin and eosin (**A–C**) and EN1 immunohistochemical staining showing positive EN1 expression (**D–F**) in different SACC histological types. Magnification, 100×, scale bar, 200 μm. **G–H** Kaplan–Meier analysis of distant metastasis-free survival (**G**) and overall survival (**H**) of SACC cases with high or low EN1 expression. **I–K** EN1 mRNA and protein expressions assessed by qPCR (**I**) and immunoblotting (**J**) in the two SACC cell lines. Quantitative results of the relative expression of EN1 protein detected by western blot (**K**). **L** Immunofluorescence staining for EN1 in SACC-83 and SACC-LM cell lines. *P < 0.05, **P < 0.01, ***P < 0.001, and ns (not significant) by t-test

To investigate the clinical significance of *EN1* expression, we performed immunohistochemical analysis of EN1 in tissue samples from 100 SACC patients. In 93 of the 100 cases, nuclear staining for EN1 was found in cells throughout the tumour, from the inner luminal cells to the outer myoepithelial cells (Fig. 2A–F). No EN1 expression was detected in normal salivary tissues. EN1 expression was also negative in the tumour cells in the three salivary ductal carcinomas, five acinic cell carcinomas, five pleomorphic adenomas, and three adenocarcinomas, NOS (Additional file 1: Figure S1).

In the 100 SACC patients, EN1 expression was not related to age, sex, histological type, or patient survival (Additional file 1: Table S3, Fig. 2G–H). However, we found higher EN1 expression in patients with metastasis compared to those without (Additional file 1: Table S3, *P* = 0.0151).

The expression of EN1 mRNA and protein was examined in two cell lines derived from SACC: SACC-LM cells, with a high rate of lung metastasis, and SACC-83 cells, with a low rate of lung metastasis [14]. Both the mRNA and protein expression of EN1 were significantly higher in SACC-LM cells than in SACC-83 cells (Fig. 2I–K**)**. Immunofluorescence results showed that EN1 was expressed in the nuclei of the two cell lines, although EN1 expression was stronger in SACC-LM cells than in SACC-83 cells (Fig. 2L).

### EN1 promotes SACC cell invasion and migration through epithelial-mesenchymal transition

**Fig. 3:**
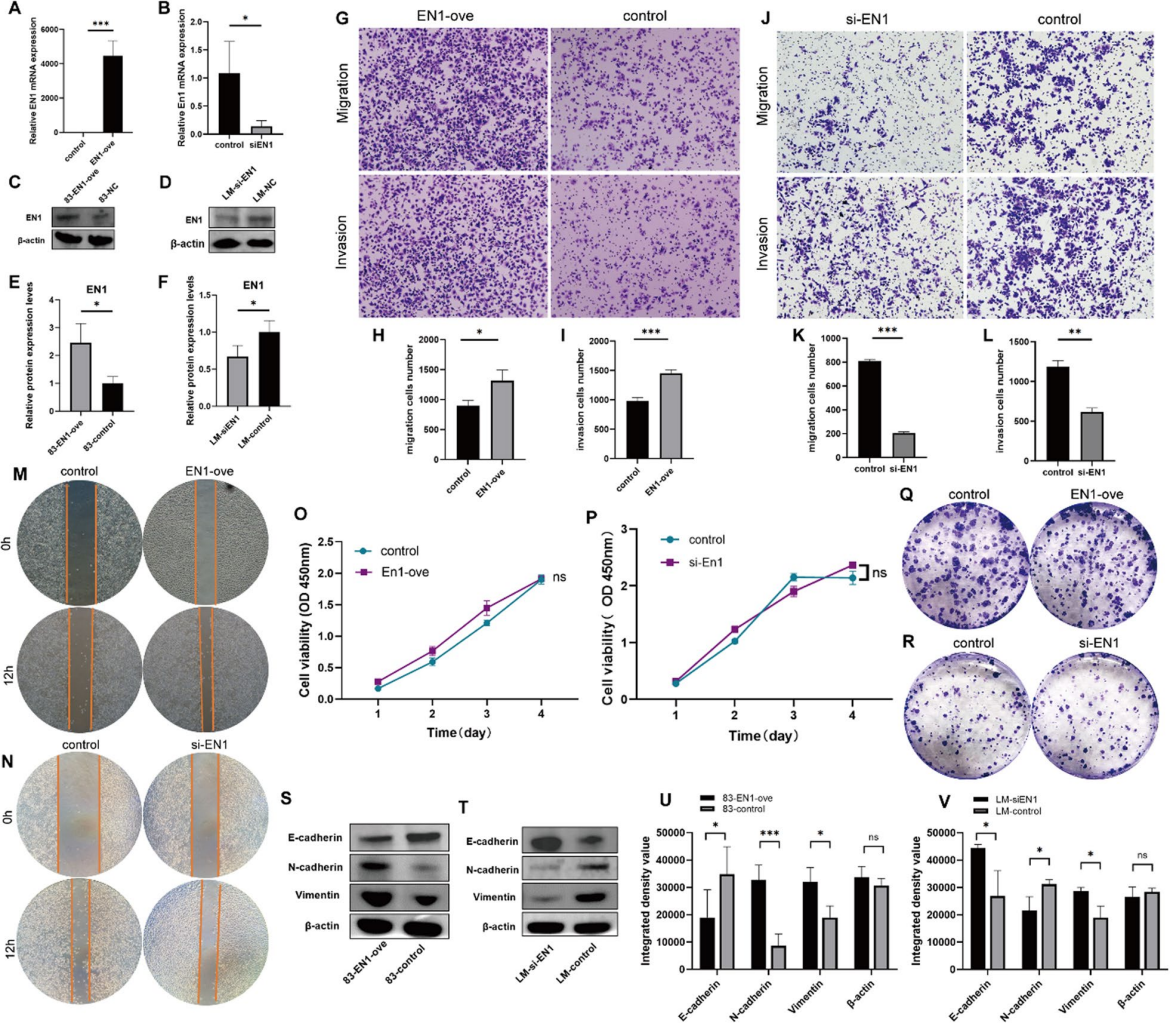
EN1 promotes SACC cell invasion and migration through EMT. **A–D** qRT-PCR (**A**,** B**) and immunoblotting (**C–F**) after *EN1* upregulation (**A**, **C**, **E**) and downregulation (**B**, **D**, **F**). Quantitative results of the relative expression of EN1 protein detected by western blot (**E–F**). **G–I** Transwell assays of the invasion and migration ability of *EN1*-overexpressing and control SACC-83 cells. **H–I** are the quantifications of the data shown in **G**. **J**–**L** Transwell assay of the invasion and migration ability of *EN1*-knockdown and control SACC-LM cells. **K–L** are the quantifications of the data shown in (**J**). **M–N** Wound-healing assays were performed to assess the migration abilities of the cells treated as indicated. **O–R** Proliferation abilities of the cells transfected as indicated were evaluated by CCK8 (**O**, **P**) and (**Q**, **R**) colony formation assays. **S–T** E-cadherin, N-cadherin, and Vimentin protein levels were analysed using western blotting. Quantitative results of the relative expression of protein detected by western blot (**U-V**). *P < 0.05, **P < 0.01, ***P < 0.001, and ns (not significant) by t-test

As there was lower expression of *EN1* in SACC-83 cells and higher expression in SACC-LM cells, we transfected an *EN1*-overexpression lentivirus into SACC-83 cells and knocked down *EN1* in SACC-LM cells using siRNA. Overexpression and knockdown efficacies were verified by qPCR and western blotting (Fig. 3A–F). EN1 overexpression resulted in an increase in the invasion and migration ability of SACC-83 cells (Fig. 3G–I, and M), and EN1 knockdown resulted in the opposite effects in SACC-LM cells (Fig. 3J–L, and N), but the proliferative ability of both cell lines did not change significantly (Fig. 3O–R). These results indicate that EN1 promoted the invasion and migration of SACC cell lines in vitro.

As epithelial-mesenchymal transition (EMT) contributes to cancer invasion, we hypothesized that the increased migration and invasion abilities of SACC induced by EN1 may be associated with the acquisition of an EMT state. Morphological changes and intercellular junctions between SACC cells in vitro were examined, and the expression of EMT markers was assessed by western blotting. In EN1-overexpressing SACC-83 cells, the epithelial biomarker E-cadherin was significantly downregulated, whereas the mesenchymal biomarkers vimentin and N-cadherin were upregulated; the opposite findings were observed in the EN1 knockdown cells (Fig. 3S and V). Taken together, these results suggest that EN1 promotes the migration and invasion of SACC cells by modulating the EMT.

### EN1 promotes SACC lung metastasis in vivo

**Fig. 4:**
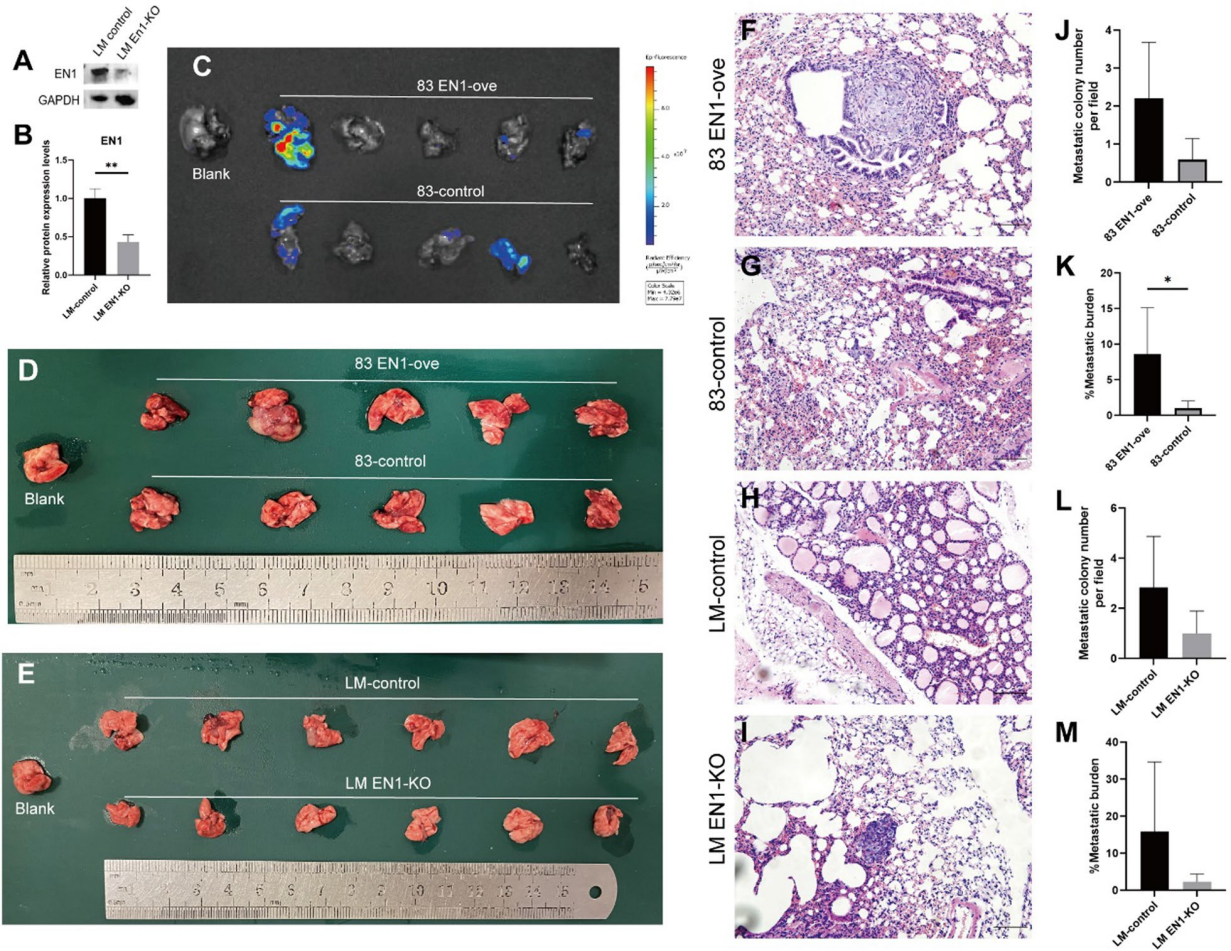
EN1 promotes SACC invasion and migration in vivo. **A–B** EN1 protein levels were examined in *EN1*-knockout SACC-LM cells by western blotting. Quantitative results of the relative expression of EN1 protein detected by western blot (**B**). **C** Fluorescence imaging of mouse lung tissue after injection of *EN1*-overexpressing (EN1-ove) and negative control SACC-83 cells was performed to evaluate lung tumour metastasis. **D** Formation of lung metastases in mice after injection of EN1-ove or negative control SACC-83 cells. **E** Formation of lung metastases in mice after injection of *EN1*-KO or negative control SACC-LM cells. **F–I** Haematoxylin and eosin staining show lung metastases in mice injected with the indicated cells. Scale bar, 100 μm. **J–M** Quantification of metastatic tumour nodules and metastatic burden in lung sections from the indicated groups. *P < 0.05, **P < 0.01, ***P < 0.001, and ns (not significant) by t-test

To examine the function of EN1 in vivo, we constructed SACC-LM *EN1*-KO cells using the CRISPR/Cas9 knockout system (Fig. 4A**–**B). We then injected EN1-ove SACC-83 cells, *EN1*-KO SACC-LM cells, and the respective negative controls into immunodeficient mice. There were no significant differences in lung tissue volume between the groups (Fig. 4C–E). However, overexpression of EN1 increased, and knocking out EN1 decreased, the lung metastasis ability of SACC cells (Fig. 4F–M).

### EN1 promotes the malignant process of SACC cells by activating the PI3K-AKT signalling pathway

**Fig. 5:**
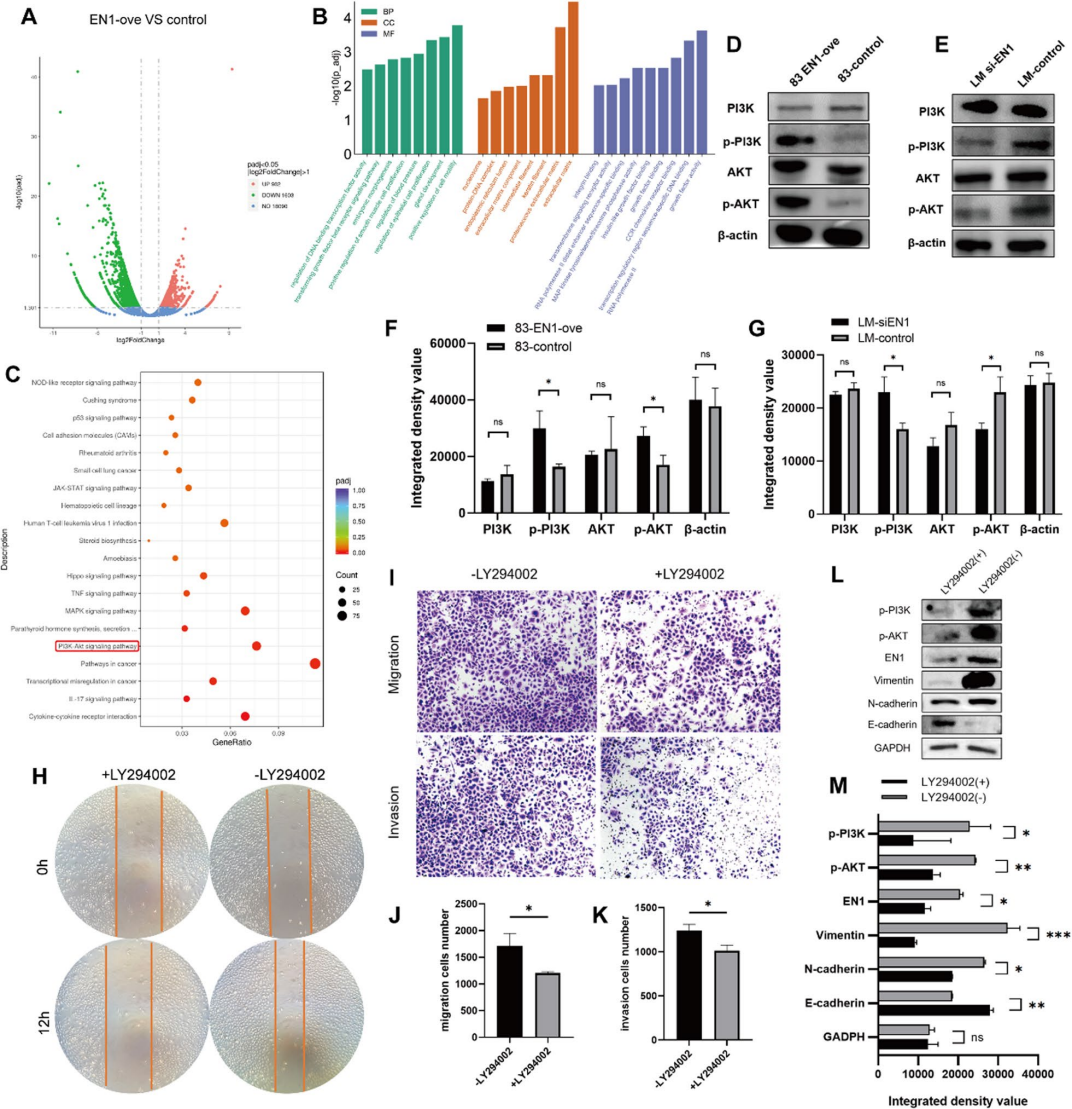
EN1 promotes the malignant process in SACC cells by activating the PI3K-AKT signalling pathway. **A** Volcano plot depicting the differentially expressed genes and their distribution in *EN1* high-expression versus control cells. The abscissa is log2 fold change, and the ordinate is -log10 (adjusted p-value). Red nodes are upregulated, and green nodes are downregulated differentially expressed genes; blue nodes are not significant. **B** Gene Ontology (GO) enrichment analysis terms for the differentially expressed genes: biological process (BP), cellular component (CC), and molecular function (MF). The abscissa is the GO term, and the ordinate is -log10 (adjusted p-value). **C** Kyoto Encyclopedia of Genes and Genomes (KEGG) pathway enrichment analysis for the differentially expressed genes. The abscissa is the gene ratio, and the ordinate is the pathway name. Node size indicates the number of genes enriched in the pathway, and node colour indicates the adjusted p-value. The PI3K-AKT pathway was enriched. **D–G** PI3K, P-PI3K, AKT, and P-AKT levels in *EN1*-overexpressing, and *EN1* knockdown and their relative control cells. **F–G** are quantitative results of the relative expression of protein detected by western blot shown in (**D–E**). **H** Wound healing assays were used to analyse EN1-ove SACC cell migration after treatment with or without LY294002. **I–K** Transwell assays were used to analyse EN1-ove SACC cell migration and invasion ability with or without LY294002 treatment. **L–M** Western blot analysis of EMT-related proteins in EN1-ove SACC cells with or without LY294002 treatment. M is the quantification of the data shown in (**L**). *P < 0.05, **P < 0.01, ***P < 0.001, and ns (not significant) by t-test

To investigate the molecular mechanism underlying the pro-metastatic effects of EN1, we performed gene expression profiling of EN1-overexpressing and negative control SACC-83 cells to identify EN1-regulated genes and pathways using RNA-seq. We identified 902 upregulated genes and 1603 downregulated genes in EN1-overexpressing cells (Fig. 5A). GO enrichment analysis showed that the significant DEGs in EN1 high-expression cells were mainly involved in the regulation of DNA transcription factor activity, cell motility, extracellular matrix formation, and growth factor activity (Fig. 5B). KEGG pathway enrichment analysis revealed that the pathways enriched by these genes were associated with the cell cycle, pancreatic cancer, Fanconi anaemia, and homologous recombination (Fig. 5C). KEGG enrichment analysis of the DEGs revealed enrichment in pathways including PI3K-AKT, TGFβ, and MAPK signalling.

The PI3K/AKT signalling pathway is involved in SACC progression [23, 24]. We examined the effects of EN1 on the PI3K/AKT signalling pathway in SACC cells. Western blot analysis revealed that the phosphorylation of PI3K and AKT increased following EN1 overexpression and reduced following EN1 knockdown (Fig. 5D–G). PI3K and AKT protein levels did not change significantly in response to changes in EN1 levels.

To examine whether EN1 contributes to malignant progression in SACC cells through activation of the PI3K/AKT pathway, we treated SACC cells with the PI3K inhibitor LY294002. Treatment with the inhibitor partially abrogated EN1 overexpression-induced metastasis and invasion (Fig. 5H–K) and reduced the expression levels of EMT-related proteins (Fig. 5L–M). These results indicated that EN1 may exert its effects on EMT in SACC via the PI3K/AKT pathway.

### Promoter methylation of EN1 and its correlation with EN1 mRNA expression

**Fig. 6:**
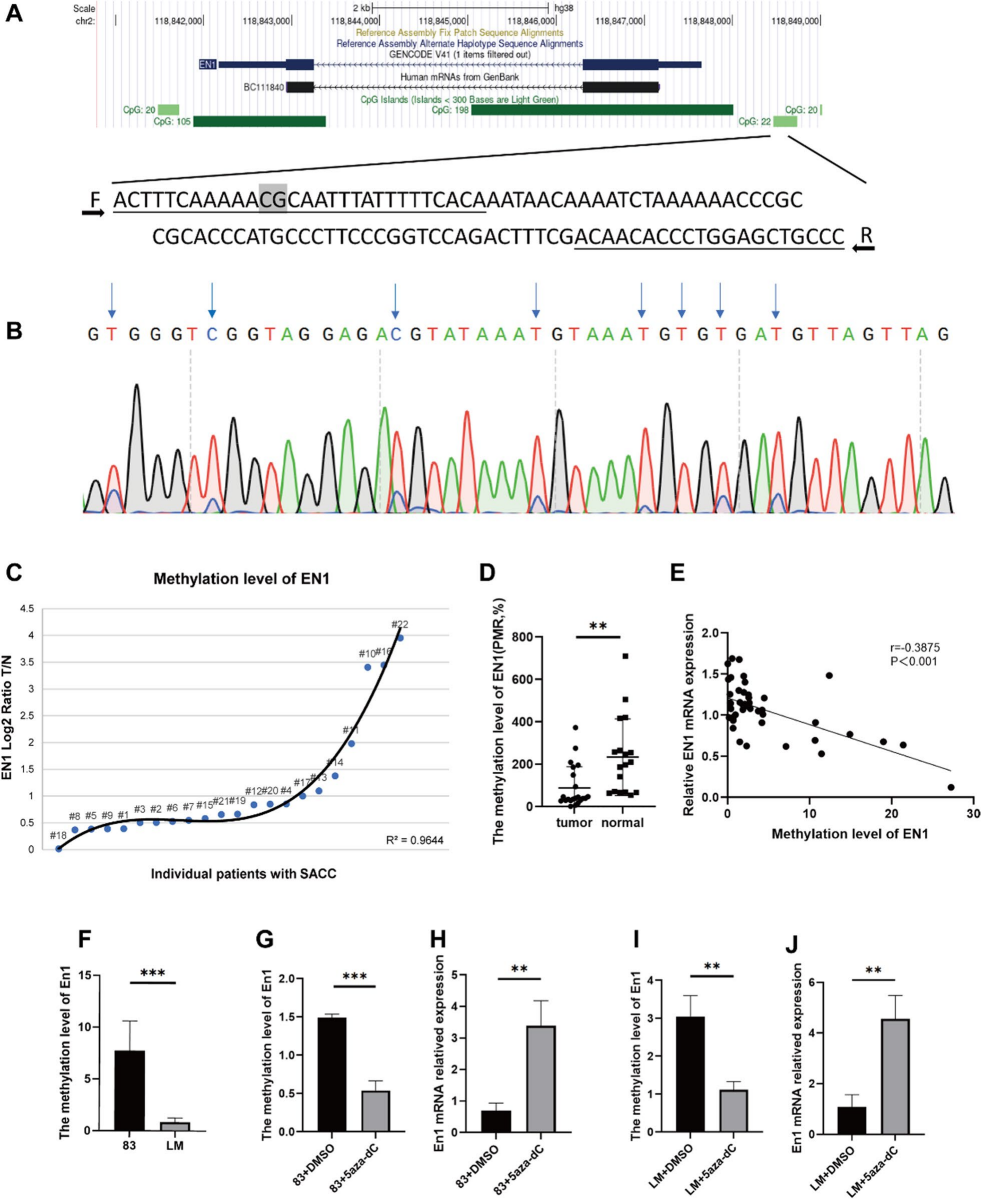
Promoter methylation status of EN1 and its correlation with EN1 mRNA expression. **A** Target sequences in the *EN1* promoter region. The genomic position of the amplified fragment was obtained from the University of California Santa Cruz genome browser using the human (GRCh38) assembly. F and R represent forward and reverse primers, respectively. CpG sites in primer sequences are highlighted in grey. **B** Sanger sequencing results. The sequence results for the fragment are shown on top. The arrows indicate converted C-T events. **C** Trendline of *EN1* promoter region hypermethylation correlated with individual tumours. **D** *EN1* methylation levels in SACC tumour and normal tissues. **E** Bioinformatic analysis of the correlation between *EN1* methylation and mRNA expression in 22 SACC samples. An inverse correlation was identified between *EN1* methylation and mRNA expression. **F** *EN1* methylation levels in SACC-83 and SACC-LM cell lines. **G–J** SACC-83 and SACC-LM cells treated with or without 5-aza-dC were evaluated for *EN1* methylation by qMSP (**G**, **I**) and *EN1* mRNA levels by qRT-PCR (**H**, **J**). **P < 0.01, ***P < 0.001, by t-test

As our results showed that EN1 is highly expressed in SACC tumours, we investigated the mechanism of EN1 upregulation. We analysed and performed a prediction analysis of the *EN1* promoter sequence and found that the *EN1* promoter harbours abundant CpG islands, indicating that *EN1* expression may be regulated by DNA methylation. Paired samples of cancerous and adjacent noncancerous tissues from 22 SACC patients (Additional file 1: Table S4) were analysed by qMSP. CpG dinucleotide methylation levels were confirmed using Sanger sequencing (Fig. 6A, B). The qMSP results confirmed significant hypomethylation of *EN1* in SACC tissues compared with paired normal tissues (R^2^ = 0.9644) (Fig. 6C), with a statistically significant difference in the overall methylation level between the tumour and normal tissues (median PMR, 0.34 vs. 0.56, *P* = 0.0027) (Fig. 6D). A negative correlation between mRNA expression and *EN1* methylation was identified (*r*=-0.3875, *P* < 0.001) (Fig. 6E).

*EN1* methylation and transcription in the SACC-83 and SACC-LM cell lines were examined. The degree of *EN1* methylation was higher in SACC-83 cells than in SACC-LM cells (Fig. 6F). After 5-aza-dC treatment, *EN1* methylation in the two cell lines was significantly reduced and *EN1* mRNA expression increased (Fig. 6G–J).

## Discussion

We identified EN1 as a key transcription factor with high expression in SACC. Immunostaining showed that EN1 was a sensitive and specific marker for SACC and might be useful in the diagnosis of SACC, especially for solid subtypes and high-grade transformation. Furthermore, EN1 overexpression was correlated with distant metastasis in SACC patients. We examined EN1 expression in SACC-83 cells and a subset of SACC cells that exhibit high lung metastasis activity (SACC-LM). Both mRNA and protein levels of EN1 were significantly higher in SACC-LM cells than in SACC-83 cells. Together, these findings suggested the potential role of EN1 in the lung metastasis of SACC.

EN1 has been reported as a potential biomarker that correlates with the progression of a variety of human cancers [7, 10, 11, 25, 26]. Bell et al. [27] observed significant hypermethylation of the *EN1* gene and EN1 protein over-expression in SACC patients, with higher expression of EN1 being associated with a lower survival rate. In patients with triple-negative breast cancer, EN1 upregulation was correlated with significantly shorter overall survival times and an increased risk of brain metastases [7]. EN1 is also specifically expressed in normal eccrine glands and focally expressed in skin tumours and sweat gland neoplasms [28]. Our findings are consistent with previous studies on EN1.

We further showed that EN1 promoted the invasion and migration of SACC cell lines in vitro and significantly increased the number of SACC lung metastases in an immunodeficient mouse model. EMT is a typical sign of tumour invasion and metastasis [29], and its involvement in SACC has also been previously reported. Our results showed that overexpression of EN1 enhanced EMT marker levels, and knockdown of *EN1* decreased their levels in SACC cells.

To explore the mechanism of EN1 in SACC, we performed RNA-sequencing on EN1-overexpressing SACC-83 and control cells and found that the DEGs were mainly enriched in the PI3K-AKT pathway. The PI3K-AKT pathway regulates the transcription, translation, and expression of oncogenes in various types of tumours; inhibits autophagic death of cancer cells; and promotes proliferation, anti-apoptosis, angiogenesis, metastasis, and drug resistance [30]. Therefore, we speculated that EN1 may play a role in the malignant progression of SACC via the PI3K-AKT pathway. The phosphorylation of PI3K and AKT increased following EN1 overexpression in SACC cells in vitro, and this effect was reversed following EN1 knockdown. A PI3K inhibitor partially abrogated EN1 overexpression-induced migration and invasion of SACC cells and reduced the expression levels of EMT-related proteins. Taken together, this indicated that EN1 might promote the migration and invasion of SACC cells by activating the PI3K-AKT signalling pathway.

Abnormal methylation of *EN1* has been reported in many malignant tumours. For example, in invasive breast cancer, *EN1* hypermethylation in the far-upstream region of the promoter was positively correlated with gene expression [31]. In contrast, the methylation level of the *EN1* promoter was negatively correlated with EN1 expression in basal-like breast tumours [32]. Bell et al. reported that *EN1* is hypermethylated in a region far upstream of the transcription initiation site, and hypermethylation is positively correlated with gene expression [2]. In our study, we found that *EN1* was hypermethylated upstream of the transcriptional start site; furthermore, hypermethylation negatively correlated with *EN1* mRNA expression levels. Inhibition of DNA methylation by 5-aza-dC increased the expression of EN1 in SACC cell lines. We found that the expression level of EN1 was not significantly related to patient prognosis. One possible hypothesis is that the expression level of EN1 is generally high, resulting in a weak correlation with prognosis. There may also be differences in the region of *EN1* in which methylation was detected compared with previous studies. This suggests that EN1 expression is influenced by epigenetic regulation.

## Conclusions

The transcription factor EN1 is a sensitive and specific marker for the differential diagnosis of SACC. EN1 promoted SACC invasion and metastasis by regulating the PI3K-Akt pathway and EMT, suggesting its potential role as a prognostic biomarker and therapeutic target for SACC.

### Supplementary Information

**Additional file 1: Figure S1.** Expression of EN1 in salivary ductal carcinoma tissue (A), adenocarcinoma, NOS (B), pleomorphic adenoma (C), and acinic cell carcinoma (D). Magnification, 200×, scale bar, 200 µm. **Table S1.** Clinical information of the 35 SACC cases analysed by RNA-seq. **Table S3.** Clinical information of the 100 SACC cases analysed for EN1 expression by immunohistochemical staining. **Table S4.** Clinical information of the 22 SACC cases analysed by qMSP.

**Additional file 2: Table S2.** Differentially expressed genes in SACC tumor compared with normal gland identified by RNA sequencing.

**Additional file 3:** Supplementary methods.

## Data Availability

No datasets were generated or analysed during the current study. Supplementary methods can be found in Supplementary Information (Additional file 3).

## Abbreviations

5-aza-dC: 5-aza-2′-deoxycytidine
DEGs: Differentially expressed genes
EMT: Epithelial-mesenchymal transition
EN1: Engrailed homeobox 1
GO: Gene ontology
KEGG: Kyoto encyclopedia of genes and genomes
PMR: Percentage of methylated reference
qMSP: Quantitative methylation-specific PCR
SACC: Salivary adenoid cystic carcinoma
