# Supplementary material for: EN1 promotes lung metastasis of salivary adenoid cystic carcinoma by regulating the PI3K-AKT pathway and epithelial-mesenchymal transition
Source: Cancer Cell Int. 2024 Jan 30;24:51. doi: 10.1186/s12935-024-03230-7 (PMC10829235; doi:10.1186/s12935-024-03230-7)
Supplement: Supplementary file 1 — Additional file 1: Figure S1. Expression of EN1 in salivary ductal carcinoma tissue (A), adenocarcinoma, NOS (B), pleomorphic adenoma (C), and acinic cell carcinoma (D). Magnification, 200×, scale bar, 200 µm. Table S1. Clinical information of the 35 SACC cases analysed by RNA-seq. Table S3. Clinical information of the 100 SACC cases analysed for EN1 expression by immunohistochemical staining. Table S4. Clinical information of the 22 SACC cases analysed by qMSP. [file 12935_2024_3230_MOESM1_ESM.docx]

Additional file


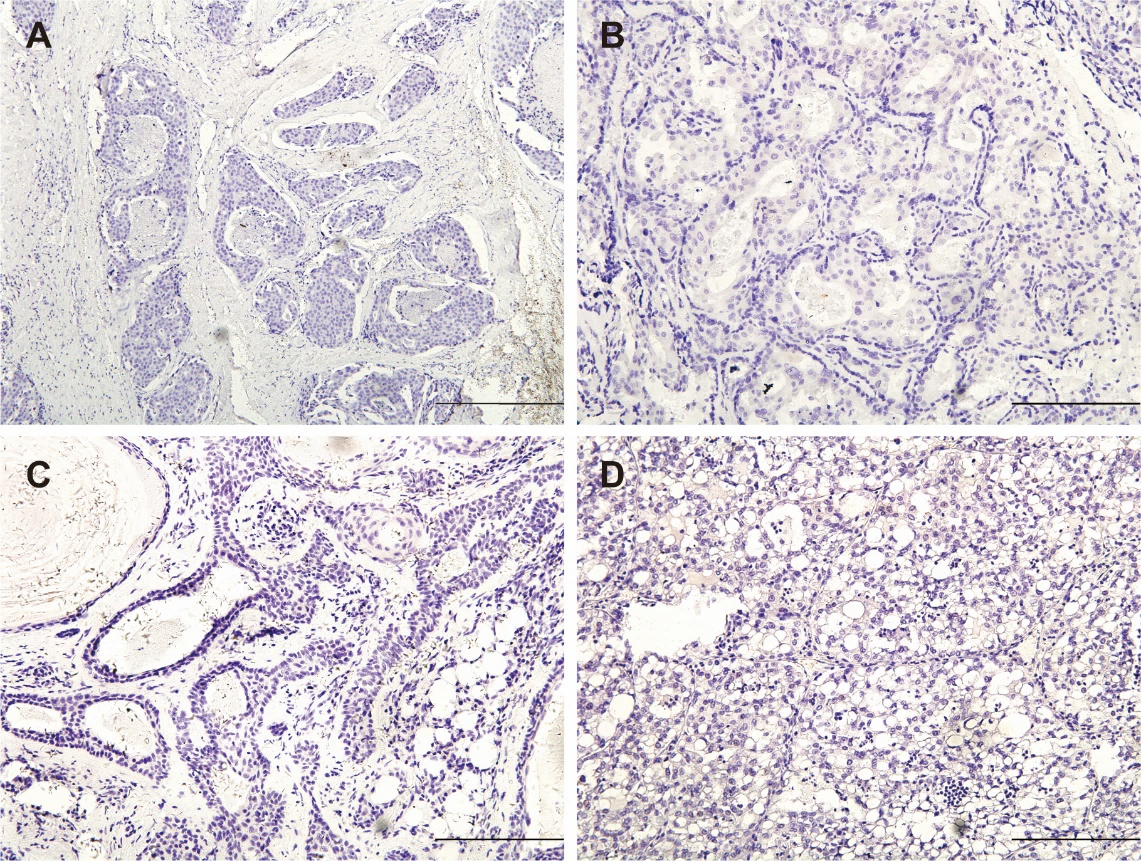


Figure S1: Expression of EN1 in salivary ductal carcinoma tissue (A), adenocarcinoma, NOS (B), pleomorphic adenoma (C), and acinic cell carcinoma (D). Magnification, 200×, scale bar, 200 µm.

Table S1 Clinical information of the 35 SACC cases analysed by RNA-seq

| **Patient ID** | **Age** | **Sex** | **Site** | **Histology type** | **Metastasis** | **Survive** |
| --- | --- | --- | --- | --- | --- | --- |
| 1 | 78 | female | sublingual gland | cribriform | no | yes |
| 2 | 59 | male | sublingual gland | cribriform | no | yes |
| 3 | 71 | female | palatal gland | tubular | no | yes |
| 4 | 67 | female | sublingual gland | solid | yes | no |
| 5 | 74 | female | submandibular gland | solid | no | yes |
| 6 | 49 | male | sublingual gland | cribriform | no | yes |
| 7 | 61 | female | sublingual gland | cribriform | no | yes |
| 8 | 63 | male | palatal gland | solid | yes | yes |
| 9 | 57 | female | palatal gland | solid | yes | no |
| 10 | 30 | female | palatal gland | solid | no | yes |
| 11 | 72 | female | sublingual gland | cribriform | no | yes |
| 12 | 56 | male | submandibular gland | solid | yes | yes |
| 13 | 26 | male | submandibular gland | solid | no | yes |
| 14 | 54 | male | palatal gland | solid | no | yes |
| 15 | 61 | male | sublingual gland | cribriform | yes | yes |
| 16 | 63 | female | sublingual gland | cribriform | no | yes |
| 17 | 40 | female | sublingual gland | solid | yes | yes |
| 18 | 42 | male | parotid gland | solid | no | yes |
| 19 | 59 | female | submandibular gland | solid | yes | no |
| 20 | 61 | male | sublingual gland | cribriform | no | yes |
| 21 | 70 | female | sublingual gland | cribriform | no | yes |
| 22 | 31 | female | submandibular gland | solid | yes | no |
| 23 | 58 | female | palatal gland | tubular | no | yes |
| 24 | 52 | female | palatal gland | cribriform | no | yes |
| 25 | 48 | female | sublingual gland | solid | no | yes |
| 26 | 78 | female | sublingual gland | cribriform | no | yes |
| 27 | 49 | male | parotid gland | cribriform | no | yes |
| 28 | 37 | female | palatal gland | tubular | yes | no |
| 29 | 67 | male | sublingual gland | cribriform | yes | no |
| 30 | 54 | male | palatal gland | cribriform | yes | yes |
| 31 | 30 | female | submandibular gland | cribriform | no | yes |
| 32 | 48 | female | palatal gland | solid | no | yes |
| 33 | 46 | male | labial gland | cribriform | no | yes |
| 34 | 24 | female | palatal gland | solid | yes | no |
| 35 | 60 | male | submandibular gland | cribriform | no | yes |

Table S3 Clinical information of the 100 SACC cases analysed for EN1 expression by immunohistochemical staining

| **Characteristics** | **Case (n=100)** | **EN1 expression** | | **P value**^c^ |
| --- | --- | --- | --- | --- |
|  |  | High^a^ | Low^b^ |  |
| **Age, y（20–78**） |  |  |  | 0.6814 |
| <45 | 33 | 30 | 3 |  |
| ≥45 | 67 | 63 | 4 |  |
| **Sex** |  |  |  | 0.7075 |
| Male | 45 | 44 | 4 |  |
| Female | 52 | 49 | 3 |  |
| **Histological type** |  |  |  | 0.4757 |
| Cribriform | 51 | 48 | 3 |  |
| Solid | 39 | 35 | 4 |  |
| Tubular | 10 | 10 | 0 |  |
| **Survival** |  |  |  | 0.6566 |
| Yes | 63 | 58 | 5 |  |
| No | 36 | 34 | 2 |  |
| **Metastasis** |  |  |  | 0.0151* |
| Metastasis | 58 | 57 | 1 |  |
| No metastasis | 42 | 36 | 6 |  |

^a^ immunohistology score 3, 4

^b^ immunohistology score 0, 1, 2

^c^ P value is based on two-sided Fisher’s exact test

Table S4 Clinical information of the 22 SACC cases analysed by qMSP

| **Patient ID** | **Age** | **Sex** | **Site** | **Histology type** | **Metastasis** | **Survive** |
| --- | --- | --- | --- | --- | --- | --- |
| 1 | 68 | female | sublingual gland | solid | no | yes |
| 2 | 60 | female | palatal gland | tubular | no | yes |
| 3 | 49 | female | sublingual gland | cribriform | yes | yes |
| 4 | 60 | female | palatal gland | cribriform | no | yes |
| 5 | 57 | male | palatal gland | cribriform | no | yes |
| 6 | 38 | male | palatal gland | cribriform | yes | yes |
| 7 | 55 | male | palatal gland | cribriform | no | yes |
| 8 | 29 | male | submandibular gland | tubular | yes | no |
| 9 | 49 | female | sublingual gland | solid | yes | no |
| 10 | 59 | male | sublingual gland | cribriform | no | yes |
| 11 | 62 | male | palatal gland | tubular | no | yes |
| 12 | 57 | male | sublingual gland | cribriform | no | yes |
| 13 | 70 | male | sublingual gland | cribriform | no | yes |
| 14 | 41 | female | parotid gland | cribriform | yes | yes |
| 15 | 31 | female | cheek area | solid | yes | no |
| 16 | 50 | female | palatal gland | tubular | no | yes |
| 17 | 23 | male | parotid gland | tubular | no | yes |
| 18 | 59 | female | sublingual gland | tubular | no | yes |
| 19 | 39 | female | palatal gland | tubular | yes | no |
| 20 | 47 | male | sublingual gland | tubular | no | yes |
| 21 | 44 | male | palatal gland | tubular | no | yes |
| 22 | 42 | male | palatal gland | cribriform | no | yes |
