# Supplementary material for: EN1 promotes lung metastasis of salivary adenoid cystic carcinoma by regulating the PI3K-AKT pathway and epithelial-mesenchymal transition
Source: Cancer Cell Int. 2024 Jan 30;24:51. doi: 10.1186/s12935-024-03230-7 (PMC10829235; doi:10.1186/s12935-024-03230-7)
Supplement: Supplementary file 3 — Additional file 3: Supplementary methods. [file 12935_2024_3230_MOESM3_ESM.docx]

Additional methods

RNA-seq and data analysis

RNA-seq and data analyses were performed as previously described[1]. Differential expression analyses of the two groups (two biological replicates per condition) were performed using the DESeq2 R package (version 1.20.0). Principal component analysis was used to assess sample clustering after variance-stabilising transformation. Functional enrichment of differentially expressed genes (DEGs) using gene sets from the Gene Ontology (GO), Kyoto Encyclopedia of Genes and Genomes (KEGG), and MSigDB hallmark gene sets was performed using clusterProfiler.

Immunofluorescence staining

Cells were seeded on coverslips, fixed, and permeabilized. The cells were then incubated with primary antibodies against EN1 (1:500; Santa Cruz Biotechnology, TX, USA), followed by incubation with TRITC-conjugated secondary antibodies (ZSGB-Bio, Beijing, China). Cells were counterstained with DAPI and imaged under a fluorescence microscope.

siRNA transfection

Cells were grown in six-well plates to 30%–50% confluence and transfected with siRNA (designed and synthesized by RiboBio, Guangzhou, China) using Lipofectamine 3000 reagent (Invitrogen, Waltham, USA) following the manufacturer's instructions. Cells were harvested after a 48-h transfection.

Cell proliferation assays

Cell proliferation was assessed using a Cell Counting Kit-8 (CCK8) (Dojindo Molecular Technologies, Kumamoto, Japan) and a clone formation assay. For the CCK8 assay, transfected cells were seeded into 96-well plates at a density of 4 × 10^3^ cells/well with 100 µL RPMI 1640 medium. After 24, 48, and 72 h, 10 µl CCK8 solution was added to each well. Absorbance was measured at 450 nm using a microplate reader (Bio-Rad Laboratories, Hercules, CA, USA).

For the clone formation assay, cells were seeded in 6-well plates at a density of 800 cells/well. After incubation for two weeks, cells were fixed with 4% formaldehyde and stained with 0.5% crystal violet. The colonies were counted and photographed. The experiments were repeated at least three times.

Cell invasion and migration assays

For invasion assays, the upper chamber of a cell culture insert (Millipore or Falcon) was precoated with Matrigel (BD Biosciences); the migration assay was performed using an insert without Matrigel. Cells were seeded at a density of 5 × 10^4^ cells/well in RPMI-1640 medium without serum in the upper chamber. The lower chamber contained RPMI-1640 medium supplemented with 10% FBS. After 18 h of incubation, cells on the upper surface of the insert were removed using a sterile cotton swab. Cells on the lower surface of the insert were fixed with 95% ethanol, stained with 1% crystal violet, and counted under a BX51 fluorescence microscope (Olympus Corporation, Tokyo, Japan) at 20x magnification. Wound healing assays were performed to assess cell migration, as described previously[2]. Each experiment was repeated at least three times.

Western blot analysis

Western blotting was performed following standard protocols. The following primary antibodies were used: anti-EN1 (1:500) and anti-β-actin (1:1000) (Santa Cruz Biotechnology); anti-GAPDH (1:5000; Biotech, Beijing, China); and anti-P-AKT, anti-AKT, anti-P-PI3K, anti-PI3K, anti-E-cadherin, anti-N-cadherin, and anti-vimentin (all 1:1000; Cell Signaling Technology). The signals were detected using an enhanced chemiluminescence kit (CW Bio, Beijing, China).

Construction of the mouse model of lung metastasis

Animal experiments were approved by the Committee on Ethics of Animal Experiments of Peking Medical University and performed in compliance with the Guide for the Care and Use of Laboratory Animals from the National Institutes of Health.

EN1-ove SACC-83 cells, EN1-knockout (EN1-KO) SACC-LM cells, and the respective negative control cells (1 × 10^6^) were injected into 8-week-old NOD/SCID mice via the tail vein (n = 6/group). One mouse died in each of the SACC-83 EN1-ove and control groups one week after injection. Bioluminescent imaging (Caliper Life Sciences, Hopkinton, USA) was used to measure lung metastasis after eight weeks. The mice were sacrificed, and the lungs were collected for haematoxylin and eosin staining to detect the number of pulmonary tumour nodules. The metastatic burden within the mouse lungs was quantified using ImageJ software.

References

1. Zhang Y, Liu X, Zhu L, Zhou Z, Cui Y, Zhou CX, Li TJ: **Notch activation promotes bone metastasis via SPARC inhibition in adenoid cystic carcinoma**. *Oral diseases* 2023.

2. Chang J, Guo C, Li J, Liang Z, Wang Y, Yu A, Liu R, Guo Y, Chen J, Huang S: **EN1 Regulates Cell Growth and Proliferation in Human Glioma Cells via Hedgehog Signaling**. *International journal of molecular sciences* 2022, **23**(3).
